# Conversion of D-fructose to 5-acetoxymethyl-2-furfural Using Immobilized Lipase and Cation Exchange Resin

**DOI:** 10.3390/molecules24244623

**Published:** 2019-12-17

**Authors:** Nhan Thanh Thien Huynh, Kyung Won Lee, Jin Ku Cho, Yong Jin Kim, Se Won Bae, Jong Shik Shin, Seunghan Shin

**Affiliations:** 1 Green Chemistry & Materials Group, Korea Institute of Industrial Technology (KITECH), 89 Yangdaegiro-gil, Ipjang-myeon, Seobuk-gu, Cheonan, Chungnam 31056, Korea; huynhthanhthiennhan@gmail.com (N.T.T.H.); c7512999@kitech.re.kr (K.W.L.); jkcho@kitech.re.kr (J.K.C.); yjkim@kitech.re.kr (Y.J.K.); swbae@kitech.re.kr (S.W.B.); 2 Department of Green Process and System Engineering, Korea University of Science and Technology (UST), 217 Gajeong-ro, Yuseong-gu, Daejeon 34113, Korea; 3 Department of Biotechnology, Yonsei University, 50 Yonsei-ro, Seodaemun-gu, Seoul 03722, Korea; enzymo@yonsei.ac.kr

**Keywords:** D-Fructose, 5-Acetoxymethyl-2-furfural (AMF), 5-Hydroxymethyl-2-furfural (HMF), trans-esterification, dehydration.

## Abstract

5-Acetoxymethyl-2-furfural (AMF) was prepared from D-fructose via 1,6-diacetylfructose (DAF) through a simple two-step reaction pathway. Immobilized enzyme (Novozym 435) was found to be the best enzymatic catalyst for the trans-esterification step (yielding 94.6% DAF). In the dehydration step, while soluble H_2_SO_4_ was found to be the best acidic catalyst (yielding 86.6% AMF), we opted to utilize heterogeneous cation exchange resin (Amberlyst 15) together with recyclable industrial solvents (1,4-dioxane) for a more sustainable AMF synthesis procedure. Although the total yield of AMF was a little lower, both the enzyme and the solid acid catalyst could be recycled for five cycles without a significant loss of activity, which has a major contribution to the cost-efficient aspect of the entire process.

## 1. Introduction

Since the 19th century, fossil fuel exploitation and refinery have been established and well developed. Currently, many aspects of the modern industry, ranging from energy and bulk chemicals to refined materials, are dependent on petroleum. Therefore, these non-renewable resources are being overly exhausted. Recent research shows great interest in using biomass resources as starters for the production of polymeric materials, organic chemicals, and fuel [1]. 5-Hydroxymethyl-2-furfural (HMF) and its derivatives are among the promising biomass-derived platform chemicals, which have the potential to become “carbon-neutral” feedstock or building blocks in a green, renewable, and sustainable industry [2,3]. Numerous studies on the synthesis of HMF from different sources of carbohydrates were conducted over the past three decades [4,5,6,7] and many of these synthesis procedures involved the dehydration of simple hexoses, such as glucose and fructose, using diverse reaction media and reaction conditions. However, reactions using polar solvents, like *N,N*-dimethylformamide (DMF) [8,9], dimethylsulfoxide (DMSO) [8,10,11], and sulfolane [12], present problems in HMF isolation while reactions with special conditions, such as microwave heating [7,13], ionic liquids [14,15,16], or supercritical-state solvents [17,18], would create a burden on the synthesis cost. In addition, HMF’s low melting point (30–34 °C) and good miscibility in both hydrophilic and hydrophobic solvents also reduce the options for purification procedures. Compared to HMF, 5-acetoxymethyl-2-furfural (AMF) [2], an ester derivative of HMF with a higher melting point (53–55 °C) and higher hydrophobicity, could be more appealing as a furan-based synthesized target. Despite the advantage, AMF has rarely been a compound of interest and was only identified as a side-product from the oxidation reaction of HMF to 2,5-furandicarboxaldehyde (FDA) [19]. Recently, Kang et al. realized that AMF has more potential as an alternative to HMF and proposed a synthesis procedure from lignocellulosic biomass via 5-chloromethyl-2-furfural (CMF) and alkylammonium acetates in biphasic solvent [20]. Furthermore, several patents on AMF’s functional group transformation were reported in the last decade [21,22,23], which makes AMF a very promising substitution for HMF since it can be converted to useful furanic monomers using similar procedures to HMF.

**Scheme 1:**
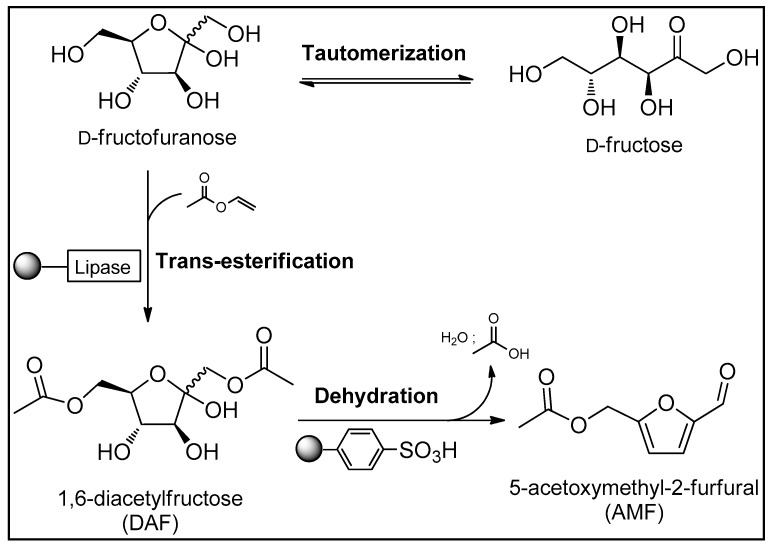
Reaction pathway for synthesis of 5-acetoxymethyl-2-furfural (AMF) from D-fructose via 1,6-diacetylfructose (DAF).

In this study, we aimed to synthesize AMF from D-fructose via an indirect pathway that involves the formation of 1,6-diacetylfructose (DAF). DAF is a di-ester derivation, which can be selectively converted from D-fructose using enzymes. Albeit briefly, the synthesis and characterization of DAF were mentioned in D’Antona et al.’s study regarding lipase-catalyzed regioselective acylation [24]. Since DAF can be selectively produced from D-fructose and easily handled, it would make an ideal intermediate for the AMF synthesis procedure. Scheme 1 explains the synthesis pathway, which consists of two reaction steps: 1) Trans-esterification of D-fructose into DAF, and 2) dehydration of DAF into AMF. This is a very new and unique approach for the synthesis of AMF since recent studies only describe a synthesis route of AMF from CMF [20,25]. Kim et al. produced AMF using a substitution reaction of CMF in DMF, with acetic acid catalyzed by a weak inorganic base. This procedure resulted in a fairly good yield of AMF, but the use of high boiling polar solvent and soluble inorganic base made the isolation of AMF quite complicated. Kang et al. greatly improved the production of AMF from CMF by combining alkylammonium acetates supported on anion exchange resin with a biphasic reaction system. This method was able to push the yield of AMF to over 90%; however, the use of CMF as substrate was not preferred, since this compound is hard to synthesize from carbohydrate sources and easily decomposes in the presence of moisture. Our approach to the AMF synthesis pathway from D-fructose via the DAF intermediate helps to avoid the problems encountered when using CMF as the starting material. The advantages of this pathway also include simple reaction steps, mild reaction conditions, environmentally friendly catalysts, and commercially available solvents. In addition, compared to the direct transformation of D-fructose into HMF, this approach, which targets the production of AMF, might help avoid the difficulties related to HMF purification since AMF can be separated from the reaction mixture by recrystallization or vacuum distillation [20]. Overall, the goal of this research was to utilize immobilized enzyme catalysts (e.g., lipase) and heterogeneous acid catalysts (e.g., Ambelyst 15) together with common industrial solvents (such as ethyl acetate, tetrahydrofuran, 1,4-dioxane) in order to establish an efficient synthesis procedure of AMF from D-fructose.

## 2. Materials and Methods

### 2.1. Materials and Equipment

D-Fructose (99%), 5-hydroxymethyl-2-furfural (HMF, 99%), 5-acetoxymethyl-2-furfural (AMF, 99%), vinyl acetate (99%), tetrahydrofuran (anhydrous, 99.9%, abbreviated as THF henceforth), 1,4-dioxane (anhydrous, 99.8%, abbreviated as dioxane henceforth), DMSO (anhydrous, 99.9%), sulfuric acid (H_2_SO_4_ 95-98%), *p*-toluenesulfonic acid monohydrate (pTSA, 98.5%), alumina (Al_2_O_3_, activated, acidic, Brockmann I), and Amberlyst 15 (hydrogen form, dry) were purchased from Sigma-Aldrich. Immobilized lipase, such as Novozym 435 and Lipozyme TL IM, were purchased from Novozymes (Denmark). Lipozyme RM IM was purchased from Sigma-Aldrich.

The main reaction system was a Carousel 12 Plus Reaction Station (Radleys, UK) equipped with a 20-mL reaction glass tube and PTFE cap. Heating, magnetic stirring, and a water-cooling system are all integrated in the Carousel 12 Plus. For scaling up the procedures, 100-mL round flasks (Schott DURAN, Wertheim am Main, Germany) were used instead of glass tubes and the reaction temperature was adjusted using an IKA-RTC basic hot plate with magnetic stirring (IKA Works GmbH & Co. KG, Staufen, Germany). The system can easily be connected with a water-cooling reflux (WiseCircu ^®^ WCH – 12 Liter, Heidelberg, Germany) for long reactions at high temperatures (e.g., dehydration). In the case of the ×600 time scale-up of trans-esterification, a 5-L jacket-type glass reactor was used. The reactor was specifically designed with two layers: The outer layer was filled with temperature-controlled water (WiseCircu ^®^ WCH – 22 Liter, Heidelberg, Germany) to heat the reaction mixture in the inner layer. The reactor was also equipped with mechanical stirring (SM 3000D, Global Lab, Gyeonggi-do, South Korea), a reflux system, and a bottom valve to collect the mixture after reaction. 

Analysis of the samples was conducted on a high-performance liquid chromatography (HPLC) system (Agilent, 1200 series equipped with diode-array detector – DAD and refractive index detector – RID) and a GC-MS system (Shimadzu GC-2010 Plus equipped with a QP2010 Ultra mass spectrometer detector). The analysis of D-fructose, DAF, AMF, and HMF in the product mixtures was performed using HPLC equipped with a Bio-rad Animex HPX-87P column with H_2_O as the eluent. D-fructose and DAF were detected by the RID detector while AMF and HMF were detected at 254 nm on the DAD detector. The formation of AMF and HMF was also verified using the GC-MS NIST 2011 database.

### 2.2. Synthetic Procedures and Analysis

1,6-Diacetylfructose (DAF): Enzymatic trans-esterification of D-fructose was performed using several types of commercially available lipase (Novozym 435, Lipozyme TL IM, and Lipozyme RM IM) as catalyst and vinyl acetate as an acyl donor. Reaction conditions were: 50 mg D-fructose, 80 µL vinyl acetate (3 equiv), 50 mg lipase, 5 mL solvent, magnetic stirring at 300 rpm, 45 °C for 5 h. After the reaction, lipase was filtered out and the filtrate was evaporated through rotary vaporization and vacuum in order to give the final product in a slightly yellow syrup form. For NMR analysis, a small portion of DAF was further purified using column chromatography. In total, 30 mg of DAF were washed through a 4-cm normal phase silica column with Et_2_O:MeOH (98:2) as the eluent. The recovered DAF was dried again in vacuum before being dissolved in an appropriate solvent for analysis. ^1^H NMR of DAF (300 MHz, CDCl_3_): 4.44–4.33 (m, 1H on C2), 4.30–4.23 (m, 2H of C5-CH_2_), 4.21–4.13 (m, 2H on C3 and C4), 4.05–4.00 (m, 2H of C2-CH_2_), 2.17–2.12 (m, 6H of 2 –COOCH_3_; major anomer: 2.14 and 2.13; minor anomer: 2.17 and 2.12) (Figure S1). ^13^C NMR of DAF (300 MHz, DMSO-d_6_): 170.83–170.47 (d, 2C in –COO– groups), 100.86 (s, C5 in the ring), 78.85 (s, C2 in the ring), 76.49 (s, C4 in the ring), 75.57 (s, C3 in the ring), 66.16 (s, C6 in –CH_2_ group), 64.62 (s, C1 in –CH_2_ group), 21.04 (m, 2C in –CH_3_ groups) (Figure S2). LC-MS analysis with API-ES positive mode +70 V (in HCOOH 0.001 M) shows m/z: 282.2 (M + H_2_O, 38%), 247.1 (M – OH^−^, 100%) (Figure S3).

5-Acetoxymethyl-2-furfural (AMF): Dehydration of DAF into AMF was performed in organic solvent using heterogeneous acid catalyst and a high temperature. Amberlyst 15, a type of cation exchange resin, was chosen as the acidic catalyst. Reaction conditions were: 50 mg DAF dissolved in 5 mL solvent, 50 mg Amberlyst 15, stirring at 300 rpm, 120 °C for 8 h. After dehydration, Amberlyst 15 was filtered out and the qualification and quantification of AMF in the reaction mixture was analyzed by HPLC and GC-MS (Figures S7 and S8). AMF was also isolated through vacuum distillation and analyzed with ^1^H NMR (300 MHz, THF-d_8_): 9.62 (s, 1H on –CHO), 7.31–7.30 (d, 1H of C4 on furan ring), 6.68–6.67 (d, 1H of C3 on furan ring), 5.13 (s, 2H of –CH_2_), 2.06 (s, 3H of –CH_3_) (Figure S4). ^13^C NMR of AMF (300 MHz, THF-d_8_): 176.91 (s, C in –CHO group), 169.23 (s, C in –COO– group), 155.57 (s, C2 in the furan ring), 155.37 (s, C5 in the furan ring), 120.62 (s, C4 in the furan ring), 112.06 (s, C3 in the furan ring), 57.27 (s, C in –CH_2_ group), 19.32 (s, C in –CH_3_ group) (Figure S5). GC-MS analysis with EI scan mode from -2.0 to +2.0 kV shows m/z: 282.2 (M - COCH_3_ + H^+^, 100%) (Figure S6).

In the reusability test, after each reaction cycle, the solid catalyst (lipase or Amberlyst 15) was filtered off using a Buchner funnel and Whatman filter paper grade 3. In the case of lipase, the immobilized enzyme was washed with THF and dried at room temperature before being reused. As for Amberlyst 15, it was washed with acetone and then dried in a vacuum oven at 25 °C before being reused.

## 3. Results and Discussion

### 3.1. Trans-Esterification of D-fructose

Lipases (triacylglycerol ester hydrolase, EC 3.1.1.3) are very commonly used in synthetic organic chemistry [24,26,27,28,29,30]. Beside the ubiquitous ability to catalyze the hydrolysis of fats and oils, lipases can catalyze enantioselective derivatization of chiral compounds, esterification, trans-esterification, and inter-esterification reactions [29]. Although lipases from different sources are able to catalyze the same reaction, their catalytic activity might be very different even under the same reaction conditions [31]. Table 1 presents the results when three types of commercially available lipases were used for the trans-esterification of D-fructose in various solvents, with vinyl acetate as the acyl donor. Since trans-esterification is a reversible reaction, the use of vinyl acetate as an acyl donor would make it become irreversible, as the by-product, vinyl alcohol, is labile and immediately converts to acetaldehyde [29]. It should be noted that lipase was chosen as the catalyst because the enzyme’s characteristics favor the trans-esterification of primary alcohol groups, which would lead to the formation of the desired di-ester derivation of D-fructose. The three particular types of immobilized lipase used for this trans-esterification were Novozym 435 (1000 PLU/g), Lipozyme TL IM (250 IUN/g), and Lipozyme RM IM (300 IUN/g). It was noticed that all three types of lipase could catalyze the trans-esterification when an ether-type solvent (THF or dioxane) was used as media for the reaction. When Novozym 435 together with THF or dioxane was used, the formation of DAF was most favorable (Entry 5,6—Table 1; 96.2% and 90.4% of DAF yield in THF and dioxane, respectively). Meanwhile, water and solvents containing –OH functional groups unsurprisingly prohibited the reaction (Entry 1,2,3—Table 1) since the –OH group of these solvents interferes with the exchange of the acetyl group from the acyl donor and primary –OH groups of D-fructose. On the other hand, solvents with a medium and low polarity index gave little or no yield of DAF (Entry 4,8,9,10,11—Table 1) because they can hardly dissolve D-fructose, thus the reaction remains stagnated when D-fructose could not be dissolved and came into contact with the immobilized lipase. It was also observed that using an excess amount of acyl donor (5 equiv. of vinyl acetate) did not improve the yield of DAF (Entry 12,13—Table 1). This screening result is consistent with the literature [24], and therefore, Novozym 435 was chosen as the source of catalyst while both THF and dioxane were used as solvents for the scaling-up procedure.

Scaling up of the trans-esterification reactions (×10 to ×600 time) was performed based on the screening results. After the scaling-up reactions, the catalyst was filtered off and recovered, the solvent was removed with rotary vaporization and vacuum, and DAF was then obtained in a yellow syrup form and stocked up as material for the dehydration step. Results in Table 2 show that scaling up the procedure had very little effect on the conversion of D-fructose as well as the formation of DAF. With some minor adjustments of the reaction time and stirring speed, we managed to achieve an 88.0% yield of DAF with 92% of D-fructose conversion (Entry 11—Table 2) in a 600 time scale-up reaction. This result indicates that trans-esterification of D-fructose with immobilized enzyme catalyst is a quite stable procedure and can be utilized for sustainable production of DAF. One major setback of this procedure is that when the concentration of the starting material was increased (from 1% *w/v* to 5% *w/v*), the yield of DAF started to decrease accordingly (a 22.9% yield drop in THF and 21.9% yield drop in dioxane was recorded). In other words, it was hard to achieve a high yield of DAF without using a large amount of solvent to fully dissolve D-fructose at the beginning state of the reaction. This was due to the low solubility of D-fructose in organic solvents and was hard to compromise. However, almost all of the solvent could be recovered and reused after the reaction.

**Figure 1:**
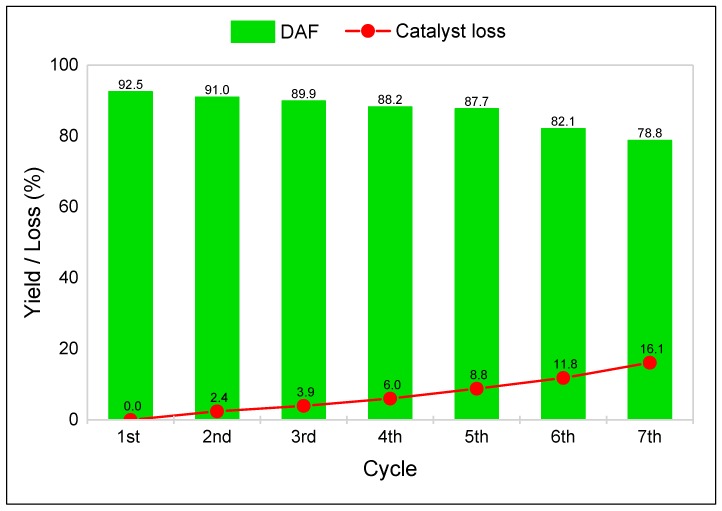
Reusability of immobilized lipase (conditions: D-fructose 500 mg, Novozym 435 500 mg (reused through all seven cycles), vinyl acetate 800 µL, THF 50 mL, 45 °C, 5 h, 300 rpm).

Experiments to investigate the reusability of the immobilized lipase were also performed in THF. After each cycle, the enzyme in the small bead form was simply filtered off, washed with THF, and dried at room temperature for 12 h before reuse. It was observed that Novozym 435 can be reused for up to five cycles with less than 9% of weight loss while still giving a stable yield of DAF (less than 5% decline in DAF yield—Figure 1). Further recycling of the enzyme led to a higher drop in DAF yield (10.4% and 13.7% drop in DAF yield at the sixth and seventh recycling circle, respectively) as well as over a 10% loss of the catalyst amount. The loss of catalyst could be explained by the partial fragmentation of catalyst beads during the long reaction and these fragments were lost in the recovery and transferring process between the cycles. As observed in Figure 1, the increment in catalyst loss correlated well with the drop in DAF yield. 

### 3.2. Dehydration of DAF

DAF could be converted to AMF by removing three molecules of water and one molecule of acetic acid under acidic conditions as depicted in Scheme S1 (Supplementary Materials). The DAF obtained from the prior trans-esterification step was dissolved in organic solvent media prior to the dehydration reaction. Based on previous studies of catalytic dehydration of sugars into furan compounds [9,14,32], Amberlyst 15 was chosen as the heterogeneous acidic catalyst for the dehydration step. Multiple experiments were conducted in order to optimize the reaction parameters, such as the reaction time, catalyst ratio, and reaction media.

**Figure 2:**
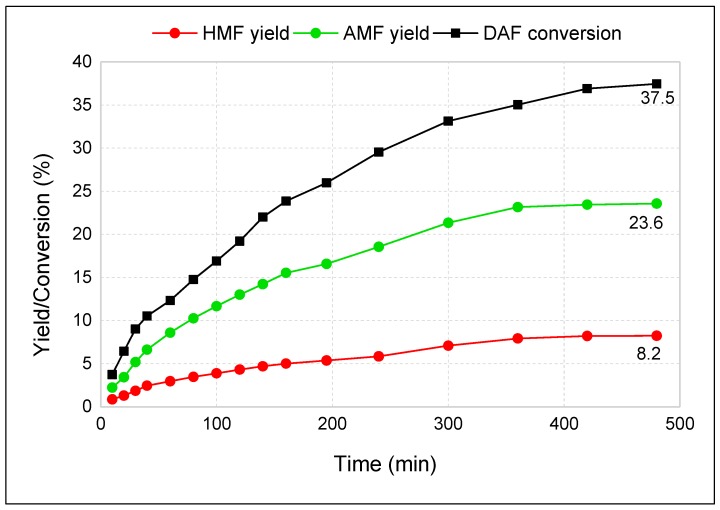
Effect of reaction time on the dehydration of DAF (Conditions: DAF 50 mg, Amberlyst 15 10 mg, 1,4-dioxane 5 mL, 120 °C, 300 rpm).

In order to study the course of the reaction, a time-dependent experiment was performed. DAF diluted in dioxane was heated at 120 °C and samples were taken at certain time intervals for HPLC analysis. It was observed that both AMF and HMF were formed under this condition, with AMF being the major product. After 8 h of reaction, we achieved the highest yield of AMF with 23.6% and 8.2% of HMF (Figure 2). Further prolongation of the reaction showed no increment of the AMF quantity. We suspected that the relatively low yield of AMF could be improved when reaction conditions, such as the solvent media or catalyst amount, were to be adjusted. In addition, the simultaneous formation of AMF and HMF in the reaction media suggested that both dehydration and hydrolysis were catalyzed in the presence of Amberlyst 15 (Scheme S2).

Since DAF is synthesized and easily dissolved in THF or dioxane, these solvents were tested to see if they are suitable for the dehydration of DAF. DMSO was also examined because AMF, unlike HMF, can be easily separated from DMSO by liquid-liquid extraction [20]. The results in Table 3 show that THF was inferior to dioxane and DMSO as a solvent for dehydration. It was understandable since THF possesses a low boiling point (66 °C) and therefore would not be very effective for the dehydration of DAF, which was performed at a much higher temperature (120 °C). Compared to the time-dependent experiment, we increased the amount of Amberlyst 15 used in this experiment to 50 mg, but the yield of AMF in dioxane was not significantly improved (23.6% compared to 29.2%). At the same time, the yield of HMF in dioxane, when increasing the amount of Amberlyst 15, was quite noticeable (from 8.2% to 15.6%). This could be an indication that the hydrolysis reaction in dioxane is a little more favorable when more acid catalyst is added.

**Figure 3:**
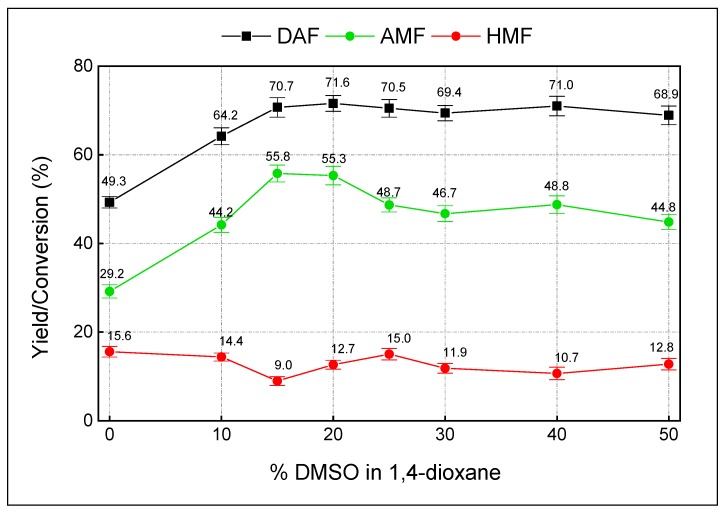
Effect of reaction media on the dehydration of DAF (Conditions: DAF 50 mg, Amberlyst 15 50 mg, solvent 5 mL, 120 °C, 8 h, 300 rpm).

The AMF yield was the highest in the case of DMSO, which has been widely and effectively used as a solvent for the dehydration of ketose-type sugars into furan compounds [33,34,35]. Several researches also mentioned that DMSO could stabilize HMF, and reduce side reactions during dehydration [36,37]. Thus, it was understandable that DMSO gave a higher total yield of HMF and AMF. On the other hand, the yield of AMF in dioxane was not so far behind when compared to in DMSO (29.2% to 38.7%), especially considering dioxane has a much lower boiling point than DMSO (101 °C compared to 189 °C). Therefore, we purposely combined DMSO and dioxane as a mixture solvent for further investigation, with the aim of maximizing the yield of AMF while limiting the formation of HMF. As the results show in Figure 3, using DMSO as a co-solvent greatly enhanced the formation of AMF while still keeping HMF at low yields. The best condition for solvent was at 15% DMSO in dioxane, which resulted in a 55.8% and 9.0% yield of AMF and HMF, respectively. It was not expected that when we increased the ratio of DMSO in dioxane to over 20%, the yield of AMF started to decline. As we mentioned that AMF and HMF were simultaneously formed during the reaction, we highly suspected that when DMSO was mixed in as co-solvent at a specific ratio, the combined solvent media had positive effects on the dehydration of DAF into furan compounds. Firstly, it was clear that the added DMSO possessed the benefit of increasing the boiling point of the reaction media, which made the combined solvent more suitable for dehydration at high temperature (120 °C). Secondly, the presence of DMSO was suggested to have a catalytic activity in the removal of H_2_O molecules from the furanose ring [33,38] as well as reducing the formation of by-products and stabilizing the formed AMF and HMF in the reaction mixture [39]. 

**Figure 4:**
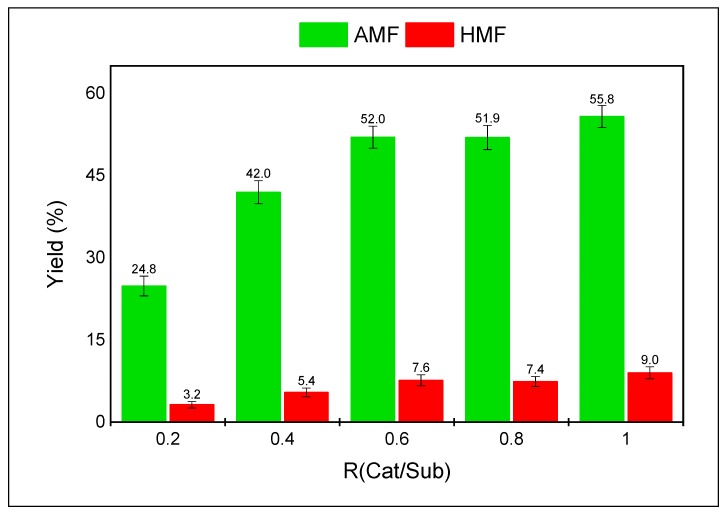
Effect of the catalyst ratio, R_Cat/Sub_, on the dehydration of DAF (Conditions: DAF 50 mg, Amberlyst 15, solvent 5 mL, 120 °C, 8 h, 300 rpm).

Since Amberlyst 15 is a cation exchange resin in the H^+^ form with a macro-reticular pore structure [40], dehydration of DAF in this case is catalyzed heterogeneously at hydrogen ion sites located throughout the resin beads. It is expected that higher amounts of catalyst during long reaction times will lead to a better yield of AMF. With that in mind, we investigated the effect of the catalyst ratio on the dehydration reaction. Upon varying the mass ratio between catalyst/substrate while maintaining the composition of reaction media at 15% DMSO in dioxane (Figure 4), it was observed that when the catalyst/substrate ratio ranged from 0.6 to 1.0 (*w/w*), the formation of AMF was significantly improved and stabilized (over 50% yield of AMF) compared to using a lower catalyst/substrate mass ratio. This means that when optimizing the procedure for sustainable production, we can slightly adjust the amount of catalyst in order to lower the cost of the reaction.

Various scaling-up dehydrations of DAF were also performed based on the best reaction media (15% DMSO in dioxane) with the ratio of Amberlyst 15/DAF (R_Cat/Sub_) at 0.6. The results in Table 4 confirm that this procedure could be effectively scaled up to 100 times without any effect on the yield of the desired AMF product (Entry 5). It was noticed that since the amount of DAF and Amberlyst 15 was increased while the solvent volume was kept at a constant value, the stirring speed had to be slightly adjusted (ranging from 300 to 500 rpm) for better heat regulation during the entire reaction course.

**Figure 5:**
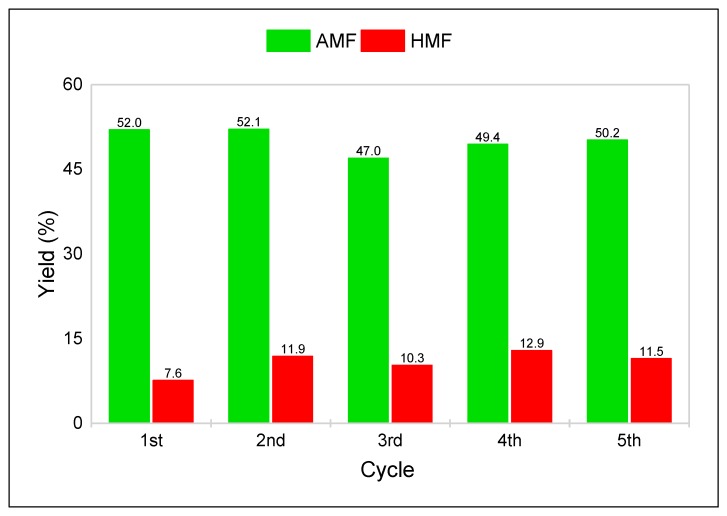
Reusability of Amberlyst 15 (Conditions: DAF 50 mg, Amberlyst 15 30 mg, solvent 5 mL, 120 °C, 8 h, 300 rpm).

The recycling of catalyst would have an impact on the cost of the reaction. Therefore, we conducted an experiment to test the reusability of Amberlyst 15 in this particular reaction since it could affect the possibility for commercialization of this procedure. After each cycle of dehydration, Amberlyst 15 was filtered, washed with acetone, and dried in a vacuum oven (25 °C) overnight before being reused. The results showed that Amberlyst 15 can be reused for up to five cycles without a remarkable change in the yield of AMF and HMF (Figure 5). It should be noted that all the cycles in the reusability test were performed using 15% DMSO in dioxane as the reaction media.

Additional experiments using different acidic catalysts were also performed as an attempt to improve the yield of AMF from DAF dehydration. Two homogeneous acid catalysts and one heterogeneous acid catalyst were used, and the results are shown in Table 5. Concentrated sulfuric acid (H_2_SO_4_) and *p*-toluenesulfonic acid (pTSA) displayed relatively high catalytic activity for the dehydration in DMSO solvent (over 73% of AMF yield, Entry 4, 5, 6, and 12—Table 5). On the other hand, alumina (Al_2_O_3_—acidic form) possessed quite low activity even when used at a higher molar ratio and longer reaction time (DAF conversion was lower than 50% and AMF yield was lower than 40%, Entry 13–20 in Table 5). Overall, DMSO proved to be a better solvent for DAF dehydration since most of the experiments performed in DMSO gave a higher conversion of DAF as well as a higher yield of AMF. When applying a mixed solvent system (15% DMSO in dioxane), only the case of Al_2_O_3_ displayed a noticeable improvement in AMF yield (entry 23—Table 5). It could be concluded that the use of a co-solvent would have little effect on dehydration with homogeneous acid catalyst. We also noticed that when the amount of homogeneous acid catalysts (from 25 mol% to 50 mol% of DAF) was increased, the yield of AMF was not much improved, or even decreased (in the case of Entry 5 and 6 in Table 5) so we conducted blank experiments to examine the stability of AMF in the presence of these acids. The results showed that when the catalyst amount was doubled, the formation of HMF from AMF was tripled (Entry 21–24 in Table 5) and a small amount of levulinic acid was detected in the reaction mixture. 

### 3.3. Two-Step Synthesis Procedure and Separation of AMF

**Scheme 2:**
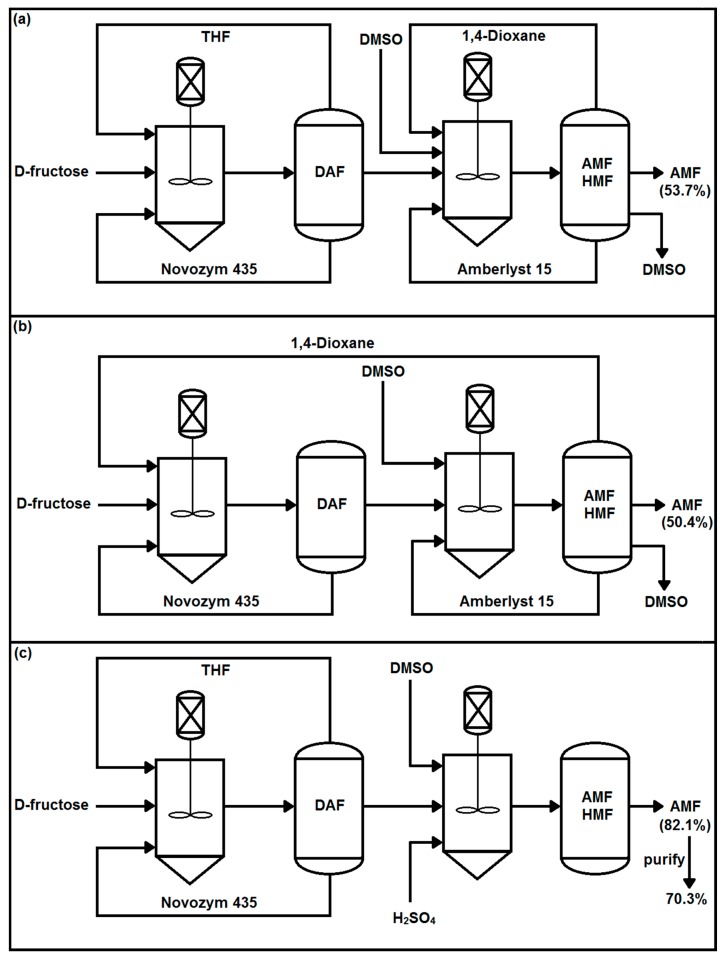
Complete synthesis procedure of AMF from D-fructose via DAF with (**a**) Novozym 435/THF followed by Amberlyst 15/DMSO-dioxane reaction system, (**b**) Novozym 435/dioxane followed by Amberlyst 15/DMSO-dioxane reaction system, and (**c**) Novozym 435/THF followed by H_2_SO_4_/DMSO reaction system.

Scheme 2 describes the complete synthesis procedure of AMF from D-fructose based on the results from both the trans-esterification step and dehydration step. The trans-esterification step can be performed efficiently in both THF (Scheme 2a) and dioxane (Scheme 2b) while the dehydration step required a mixture of DMSO in dioxane as the reaction media. In both cases, the final yield of AMF was similar (53.7% and 50.4%, respectively) and the solvents (THF, dioxane) can be recovered through rotary vaporization and reused after distillation. After removing most of the solvent, the final reaction mixture can be distilled under vacuum at 106 to 109 °C to achieve AMF with 99% purity (the analysis results of AMF are given in the Supplementary Material). 

When combining the enzymatic trans-esterification and the acidic homogeneous dehydration, the yield of AMF was significantly boosted (Scheme 2c). However, the purification step for AMF in this case is more complicated since H_2_SO_4_ needs to be neutralized and a larger amount of DMSO needs to be removed. The reaction mixture was first diluted in ethyl acetate, washed with aqueous NaOH (×3 times), and then water (×5 times) to transfer H_2_SO_4_ and DMSO to the aqueous phase while retaining AMF in the organic phase. Ethyl acetate was then evaporated, and the remaining product was further distilled under vacuum to achieve AMF (70.3% yield with 99% purity). Although this procedure resulted in a higher yield of the desired product, the catalyst and solvent for the dehydration step could only be discarded after the reaction. Thus, it was less favorable to use this as a green and sustainable manufacturing method for AMF.

## 4. Conclusions

In this research, a simple two-step synthesis of AMF from D-fructose was investigated. D-fructose was purposely converted to DAF using lipase and then dehydrated to achieve AMF. Both synthesis steps utilized commercially available catalyst, such as Novozym 435 (immobilized Lipase B) and Amberlyst 15, together with common industrial solvents in order to establish a sustainable manufacturing procedure of AMF from D-fructose. In addition, the organic solvents (THF, dioxane) could be recovered and both catalysts reused for five cycles, which is a big advantage when determining the cost of AMF production. To the best of our knowledge, there has been no precedent study that utilizes both immobilized enzyme and cation exchange resin for the synthesis of 5-acetoxymethyl-2-furfural.

## Figures and Tables

**Table 1:** D-Fructose trans-esterification in various solvents ^a^.

Entry	Solvent	DAF yield (%)		
		Lipozyme TL IM	Lipozyme RM IM	Novozym 435
1	H_2_O	0.0	0.0	0.0
2	MeOH	0.0	0.0	0.0
3	EtOH	0.0	0.0	0.0
4	Acetone	7.4 ± 1.2	0.0	12.7 ± 2.4
5	THF	44.3 ± 2.7	30.1 ± 2.3	96.2 ± 2.8
6	Dioxane	37.4 ± 3.2	22.5 ± 1.8	90.4 ± 2.5
7	DMSO	0.0	0.0	0.0
8	Ethyl acetate	9.1 ± 2.1	0.0	14.4 ± 2.2
9	MIBK	5.7 ± 1.9	0.0	9.7 ± 1.8
10	Hexane	0.0	0.0	0.0
11	Dichloromethane	0.0	0.0	0.0
12 ^b^	THF	-	-	95.5 ± 2.9
13 ^b^	Dioxane	-	-	92.2 ± 2.3

^a^ Conditions: D-fructose 50 mg, immobilized lipase 50 mg, vinyl acetate 80 µL (3 equiv.), solvent 5 mL, 45 °C, 5 h, 300 rpm. ^b^ Using 5 equiv. of vinyl acetate.

**Table 2:** Scale up trans-esterification of D-fructose ^a^.

Entry	Solvent	D-Fructose (g)	D-Fructose Conversion (%)	DAF Yield (%)	DAF Selectivity (%) *
1	THF	0.5	100	94.6	94.6
2	THF	1.0	98.3	90.1	91.6
3	THF	1.5	90.1	82.8	91.9
4	THF	2.0	83.5	77.3	92.6
5	THF	2.5	76.7	71.7	93.5
6	Dioxane	0.5	100	91.3	91.3
7	Dioxane	1.0	95.7	86.7	90.6
8	Dioxane	1.5	88.8	80.2	90.3
9	Dioxane	2.0	79.9	75.4	94.4
10	Dioxane	2.5	73.4	69.4	94.6
11 ^b^	THF	30.0	92.0	88.0	95.6

^a^ Conditions: m_D-fructose_ = m_Novozym 435_, vinyl acetate (3 equiv.), solvent 50 mL, 45 °C, 5 h, 300 rpm; ^b^ Conditions: D-fructose 30 g, Novozym 435 30 g, vinyl acetate 46 mL (3 equiv.), THF 3 L, 45 °C, 6 h, 500 rpm; * DAF selectivity was calculated using this formula: $DAF\;selectivity\;\left(\%\right)=\;\frac{DAF\;yield\;\left(\%\right)}{D-fructose\;conversion\;\left(\%\right)}\;\times 100$.

**Table 3:** DAF dehydration in different solvents.

Solvent	DAF Conversion (%)	AMF Yield (%)	HMF Yield (%)
THF	12.9 ± 1.0	6.1 ± 1.6	3.8 ± 0.8
Dioxane	51.2 ± 1.2	29.2 ± 2.1	15.6 ± 1.7
DMSO	59.2 ± 1.4	38.7 ± 2.4	14.9 ± 1.9

Conditions: DAF 50 mg, Amberlyst 15 50 mg, solvent 5 mL, 120 °C, 8 h,300 rpm.

**Table 4:** Scaling-up dehydration of DAF using 15% DMSO in dioxane as solvent.

Entry	DAF (g)	DAF Conversion (%)	AMF Yield (%)	HMF Yield (%)
1	0.5	69.9	54.7	9.8
2	1.0	71.2	54.2	11.2
3	2.0	73.8	56.1	13.7
4	4.0	74.7	55.4	15.5
5	5.0	74.9	57.9	15.8

Conditions: R_Cat/Sub_ = 0.6, solvent 50 mL, 120 °C, 8 h, 300–500 rpm.

**Table 5:** Dehydration of DAF using various acid catalysts.

Entry	Catalyst - mol% of DAF	Solvent	Time (h)	DAF Conversion (%)	AMF Yield (%)	HMF Yield (%)
1	H_2_SO_4_-10%	Dioxane	2	69.1 ± 2.3	49.9 ± 1.9	15.2 ± 2.5
2	H_2_SO_4_-25%	Dioxane	2	84.6 ± 1.8	61.7 ± 1.5	17.6 ± 2.2
3	H_2_SO_4_-50%	Dioxane	2	94.5 ± 2.2	59.4 ± 2.8	22.7 ± 3.4
4	H_2_SO_4_-10%	DMSO	2	88.2 ± 1.2	73.2 ± 2.7	11.7 ± 1.4
5	H_2_SO_4_-25%	DMSO	2	98.4 ± 1.6	86.6 ± 1.7	10.4 ± 1.3
6	H_2_SO_4_-50%	DMSO	2	100 ± 0.0	78.1 ± 3.4	14.9 ± 3.8
7	pTSA-10%	Dioxane	4	66.6 ± 1.1	49.3 ± 2.0	12.4 ± 1.8
8	pTSA-25%	Dioxane	4	85.2 ± 1.3	60.9 ± 1.0	19.4 ± 2.4
9	pTSA-50%	Dioxane	4	97.0 ± 0.9	63.7 ± 3.0	24.5 ± 3.3
10	pTSA-10%	DMSO	4	66.8 ± 2.4	52.2 ± 2.5	10.3 ± 1.2
11	pTSA-25%	DMSO	4	83.4 ± 2.7	69.1 ± 2.0	10.2 ± 1.2
12	pTSA-50%	DMSO	4	100 ± 1.7	73.0 ± 2.3	19.9 ± 1.6
13	Al_2_O_3_-10%	Dioxane	8	29.2 ± 1.4	22.8 ± 1.1	3.9 ± 0.6
14	Al_2_O_3_-25%	Dioxane	8	30.7 ± 1.7	23.9 ± 1.5	3.8 ± 1.7
15	Al_2_O_3_-50%	Dioxane	8	34.7 ± 1.3	26.1 ± 1.4	4.4 ± 0.8
16	Al_2_O_3_-100%	Dioxane	8	42.8 ± 2.4	31.6 ± 2.0	6.9 ± 1.5
17	Al_2_O_3_-10%	DMSO	8	33.1 ± 1.4	24.5 ± 1.5	5.2 ± 1.0
18	Al_2_O_3_-25%	DMSO	8	33.8 ± 0.9	24.9 ± 1.3	5.0 ± 1.2
19	Al_2_O_3_-50%	DMSO	8	40.9 ± 1.9	28.6 ± 1.5	7.0 ± 1.1
20	Al_2_O_3_-100%	DMSO	8	49.6 ± 1.5	39.9 ± 2.3	6.2 ± 1.9
21	H_2_SO_4_-25%	15%DMSO in dioxane	2	99.1 ± 0.7	85.3 ± 0.9	11.0 ± 1.3
22	pTSA-50%	15%DMSO in dioxane	4	96.6 ± 1.4	76.2 ± 2.9	14.8 ± 2.2
23	Al_2_O_3_-100%	15%DMSO in dioxane	8	60.9 ± 2.1	48.3 ± 1.8	6.6 ± 1.5
24*	H_2_SO_4_-25%	DMSO	2	-	95.7	3.9
25*	H_2_SO_4_-50%	DMSO	2	-	83.4	12.3
26*	pTSA-25%	DMSO	4	-	94.4	4.3
27*	pTSA-50%	DMSO	4	-	82.1	13.9

Conditions: DAF 50 mg, acid catalyst, solvent 5 mL, 120 °C, 300 rpm; * blank experiment: AMF 50 mg, acid catalyst, solvent 5 mL, 120 °C, 300 rpm.

## Supplementary Materials

Analysis results are available for HPLC, LC-MS, GC-MS, and NMR. Schemes for plausible mechanism from DAF to AMF and for a suggested pathway for the formation of AMF and HMF are also provided.
