# Supplementary material for: Conversion of D-fructose to 5-acetoxymethyl-2-furfural Using Immobilized Lipase and Cation Exchange Resin
Source: Molecules. 2019 Dec 17;24(24):4623. doi: 10.3390/molecules24244623 (PMC6943744; doi:10.3390/molecules24244623)
Supplement: Supplementary file 1 [file molecules-24-04623-s001.pdf]

molecules

Supplementary materials

Conversion of D-fructose to 5-acetoxymethyl-2-furfural using immobilized lipase and cation exchange resin

Nhan Thanh Thien Huynh [1,2], Kyung Won Lee [1,3], Jin Ku Cho [1,2], Yong Jin Kim [1,2], Baekjin Kim [1,2], Jong Shik Shin [3], and Seunghan Shin [1,2,*]

[1] Green Chemistry & Engineering R&D Department, Korea Institute of Industrial Technology (KITECH), 89 Yangdaegiro-gil, Ipjang-myeon, Seobuk-gu, Cheonan, Chungnam 31056, Korea
[2] Department of Green Process and System Engineering, Korea University of Science and Technology (UST), 217 Gajeong-ro, Yuseong-gu, Daejeon 34113, Korea
[3] Department of Biotechnology, Yonsei University, 50 Yonsei-ro, Seodaemun-gu, Seoul 03722, Korea
* Correspondence: shshin@kitech.re.kr. Tel: +82-41-5898-422. Fax: +82-41-5898-580

Received: date; Accepted: date; Published: date

1. Characterization methods:

1.1 Nuclear Magnetic Resonance (NMR):

NMR analysis was performed on a Bruker Spectrospin 300 (Bruker Corporation, Germany). Figure S1 shows ^{1}H NMR of DAF (300 MHz, $CDCl_3$): 4.44-4.33 (m, 1H on C2), 4.30-4.23 (m, 2H of C5-CH_2), 4.21-4.13 (m, 2H on C3 and C4), 4.05-4.00 (m, 2H of C2-CH_2), 2.17-2.12 (m, 6H of 2 –$COOCH_3$; major anomer: 2.14 and 2.13; minor anomer: 2.17 and 2.12). ^{13}C NMR of DAF (300 MHz, DMSO-d_6): 170.83-170.47 (d, 2C in –COO– groups), 100.86 (s, C5 in the ring), 78.85 (s, C2 in the ring), 76.49 (s, C4 in the ring), 75.57 (s, C3 in the ring), 66.16 (s, C6 in –CH_2 group), 64.62 (s, C1 in –CH_2 group), 21.04 (m, 2C in –CH_3 groups) (Figure S2).

AMF was also analyzed with NMR and Figure S4 shows ^{1}H NMR of AMF (300 MHz, THF-d_8): 9.62 (s, 1H on –CHO), 7.31-7.30 (d, 1H of C4 on furan ring), 6.68-6.67 (d, 1H of C3 on furan ring), 5.13 (s, 2H of –CH_2), 2.06 (s, 3H of –CH_3). ^{13}C NMR of AMF (300 MHz, THF-d_8): 176.91 (s, C in –CHO group), 169.23 (s, C in –COO– group), 155.57 (s, C2 in the furan ring), 155.37 (s, C5 in the furan ring), 120.62 (s, C4 in the furan ring), 112.06 (s, C3 in the furan ring), 57.27 (s, C in –CH_2 group), 19.32 (s, C in –CH_3 group) (Figure S5).

1.2 High performance liquid chromatography (HPLC):

HPLC analysis of the dehydration products was performed on an Agilent 1200 series equipped with a Bio-Rad Animex HPX-87P column and an RID detector (at 254 nm). H_2O was used as mobile phase and the flow rate was adjusted at 0.6 mL/min. The retention times for AMF and HMF are 54.2 min. and 29.3 min. respectively (shown in figure S7).

1.3 Liquid chromatography combined with mass spectroscopy (LC-MS):

LC-MS analysis of the DAF was performed on an Agilent 1260 Infinity II series equipped with an InfinityLab LC/MSD mass spectrometer detector. HCOOH 0.001 M in H_2O was used as mobile phase and the flow rate was adjusted at 0.5 mL/min. DAF was ionized in API-ES positive mode and the mass spectrum was shown in Figure S3.

1.4 Gas chromatography combined with mass spectroscopy (GC-MS):

GC-MS analysis of the dehydration products was performed on a Shimadzu GC-2010 Plus system equipped with QP2010 Ultra mass spectrometer detector. AMF and HMF formation (Figure S8) is verified by comparing spectrum with GC-MS NIST 2011 database.

2. Analysis results:

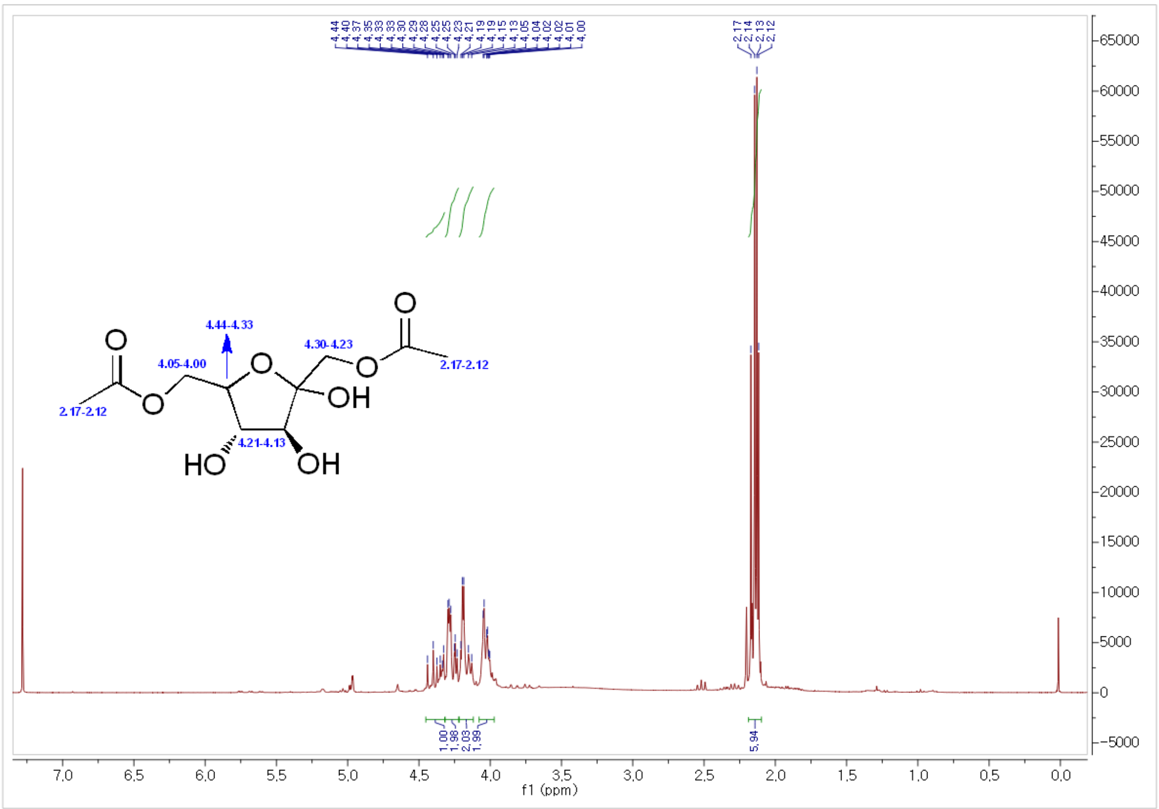

Figure S1. ^{1}H NMR analysis of DAF

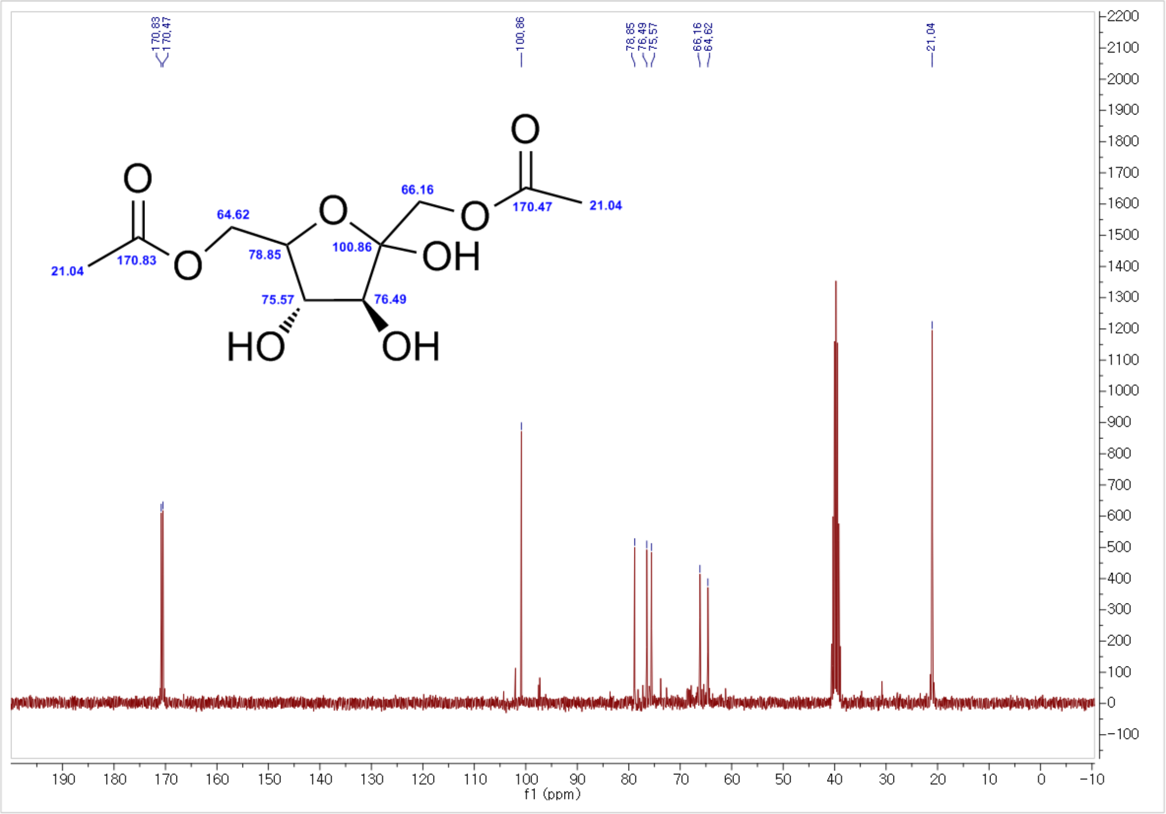

Figure S2. ^{13}C NMR analysis of DAF

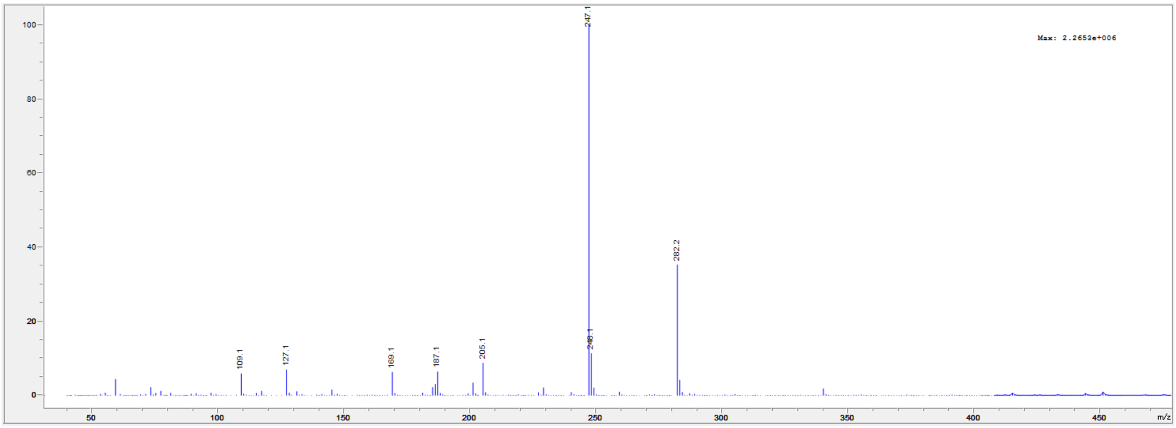

Figure S3. Mass spectrum of DAF

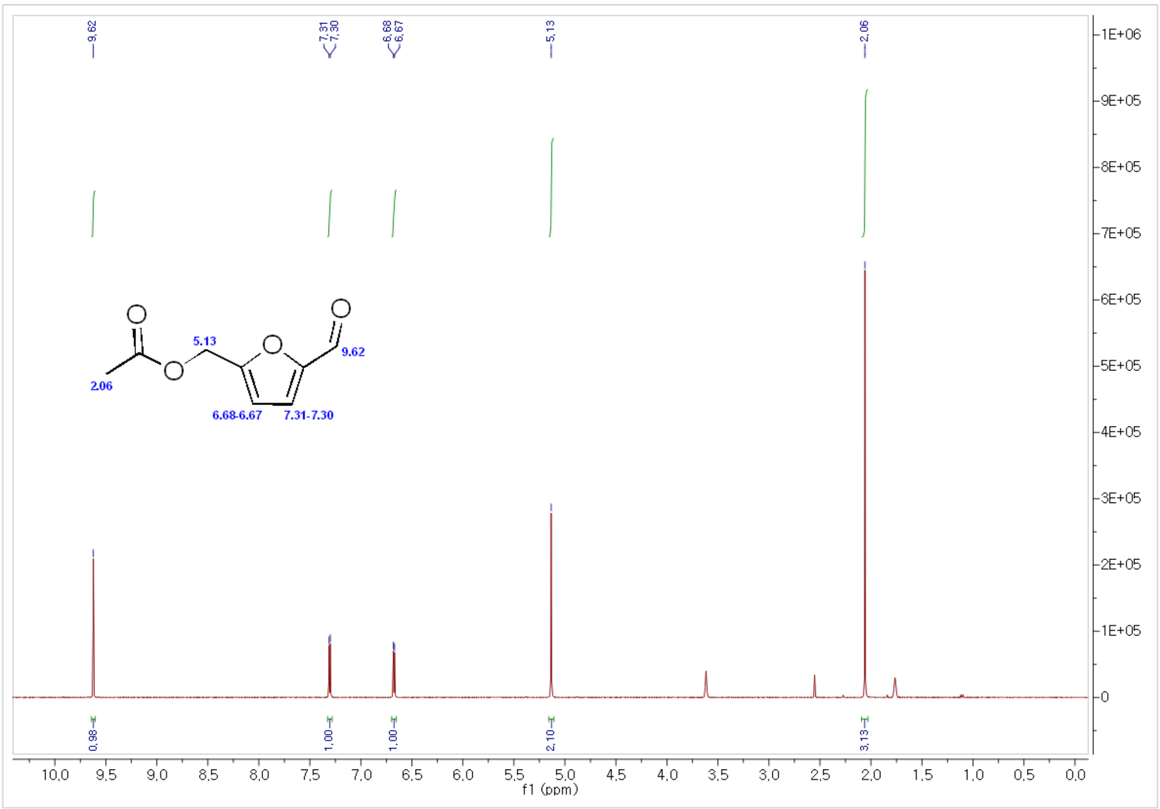

Figure S4. ^{1}H NMR analysis of AMF

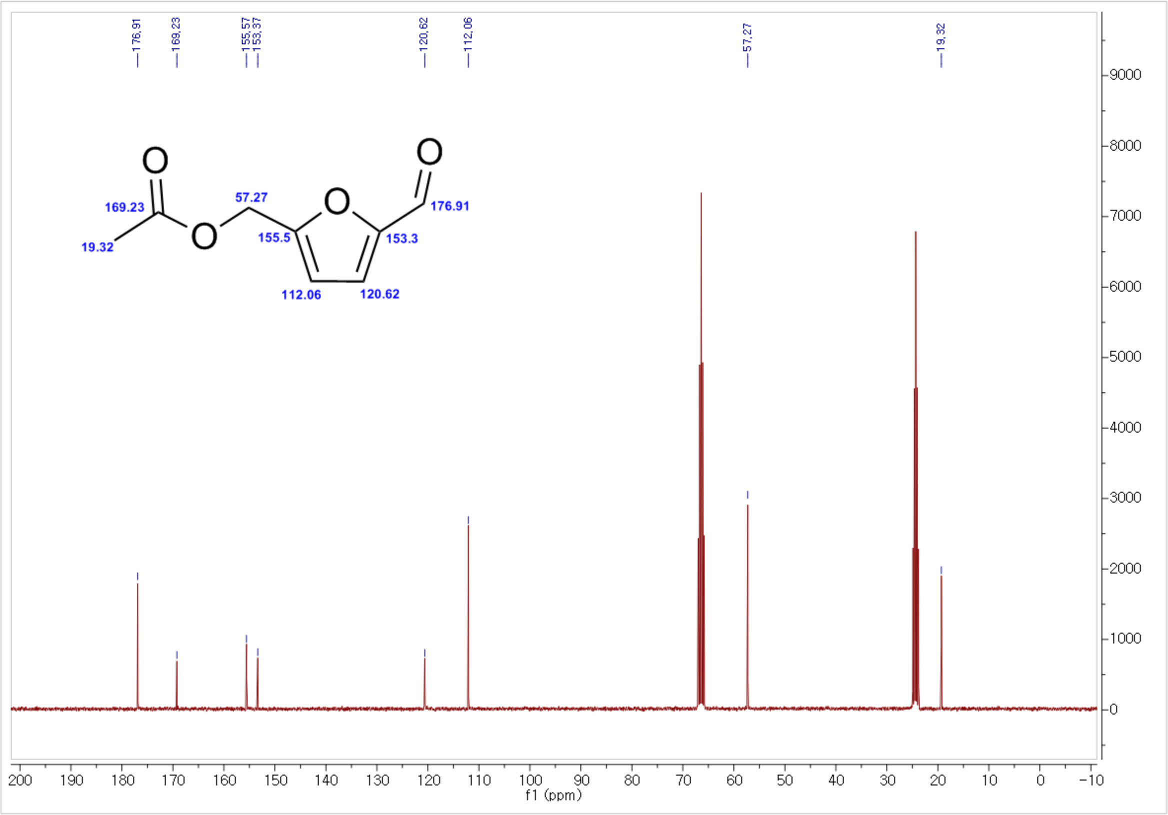

Figure S5. ^{13}C NMR analysis of AMF

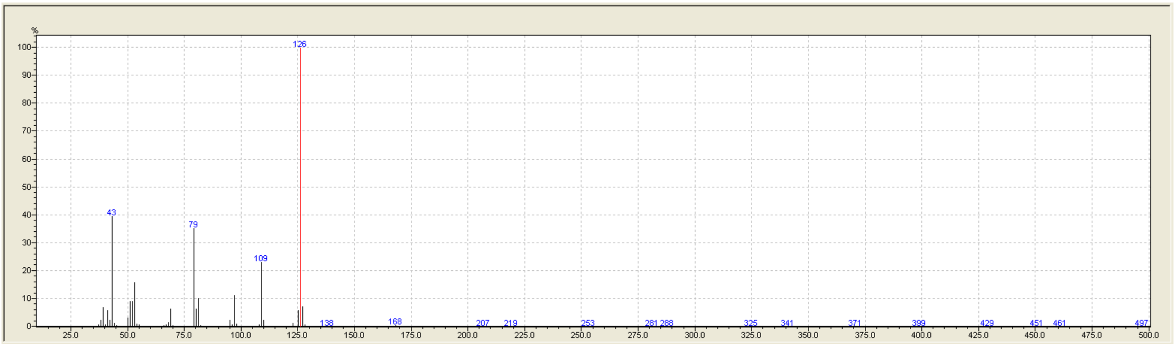

Figure S6. Mass spectrum of AMF

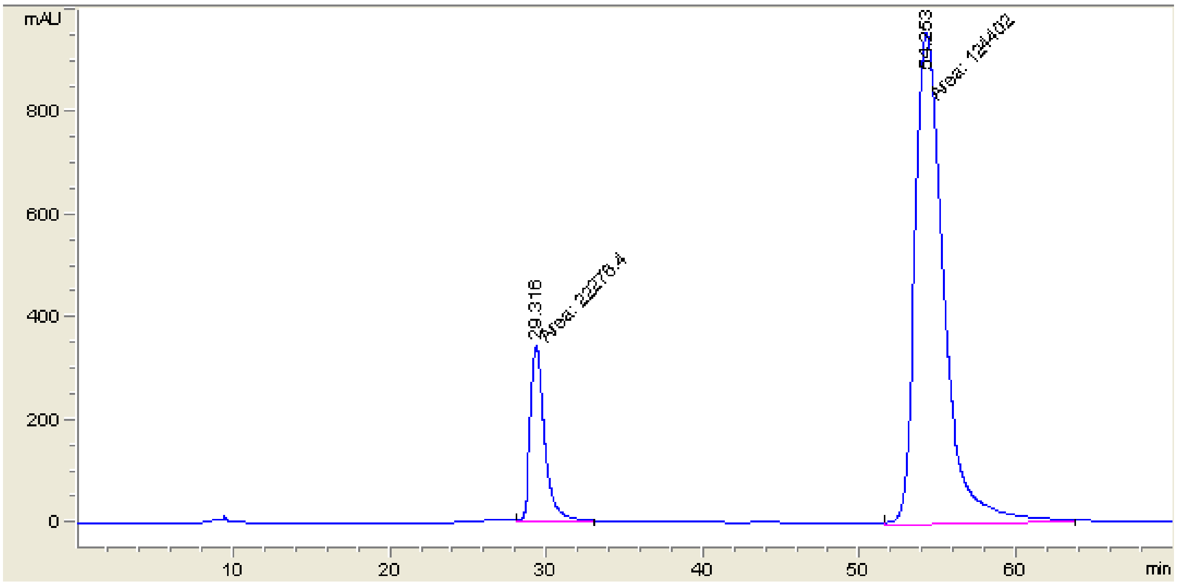

Figure S7. HPLC analysis of AMF and HMF

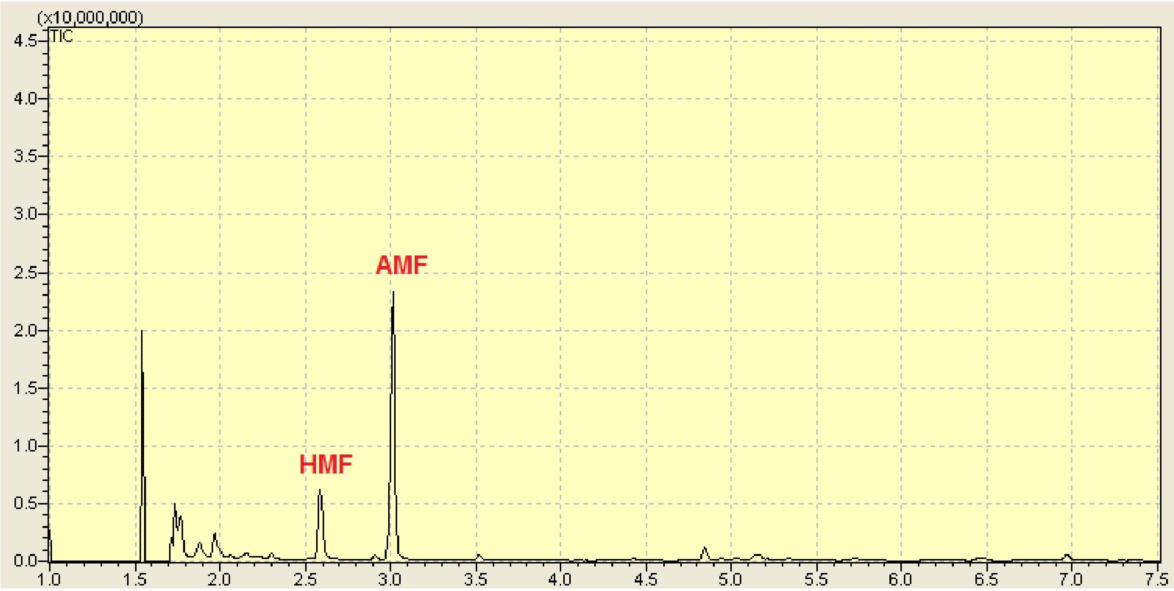

Figure S8. GC-MS analysis of AMF and HMF

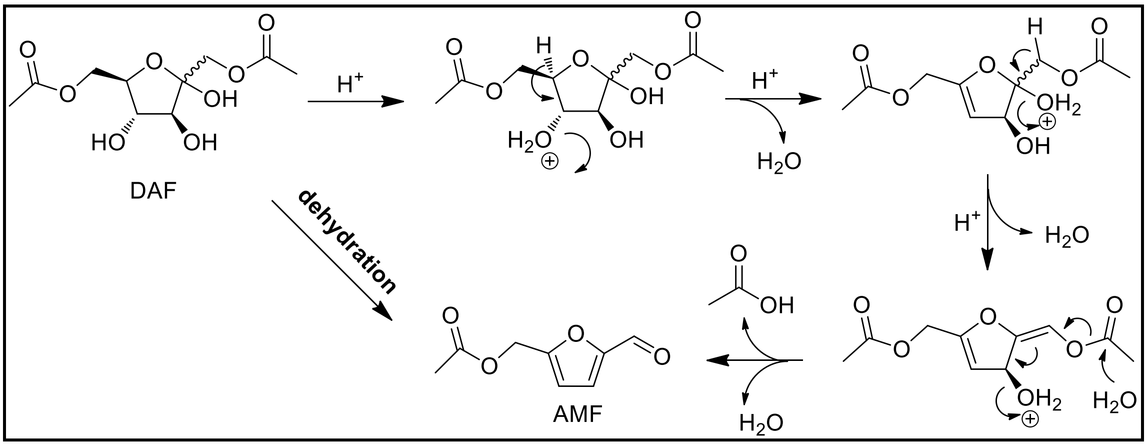

Scheme S1. Plausible mechanism for the dehydration of DAF to AMF under acidic conditions

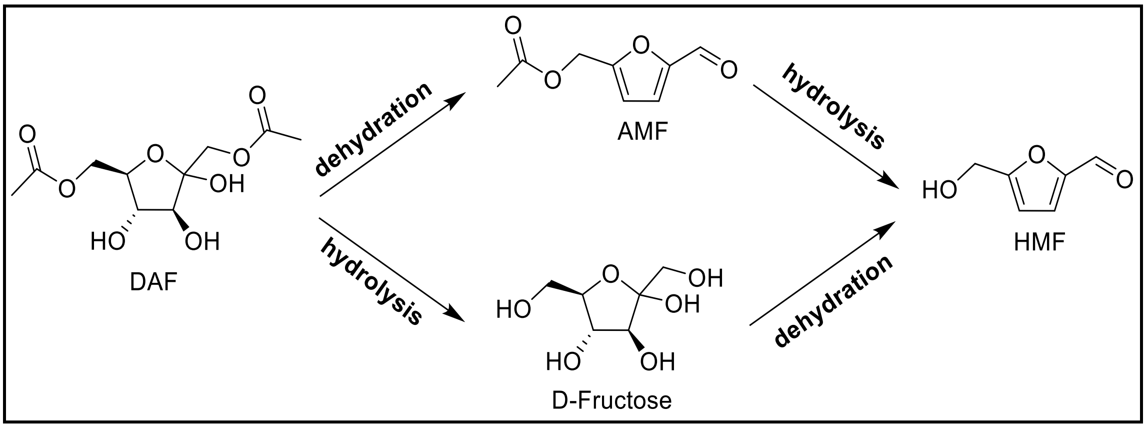

Scheme S2. Suggested pathway for the formation of AMF and HMF in the dehydration of DAF
